# Qualitative and Quantitative DNA- and RNA-Based Analysis of the Bacterial Stomach Microbiota in Humans, Mice, and Gerbils

**DOI:** 10.1128/mSystems.00262-18

**Published:** 2018-11-20

**Authors:** Philipp Wurm, Elisabeth Dörner, Christina Kremer, Julia Spranger, Cynthia Maddox, Bettina Halwachs, Ute Harrison, Thomas Blanchard, Rainer Haas, Christoph Högenauer, Gregor Gorkiewicz, W. Florian Fricke

**Affiliations:** a Institute of Pathology, Medical University Graz, Graz, Austria; b Theodor Escherich Laboratory for Medical Microbiome Research, Medical University of Graz, Graz, Austria; c Universität Hohenheim, Stuttgart, Germany; d BioTechMed Interuniversity Cooperation, Graz, Austria; e Max von Pettenkofer-Institut, Ludwig-Maximilians-Universität München, Munich, Germany; f Division of Gastroenterology and Hepatology, Department of Internal Medicine, Medical University of Graz, Graz, Austria; g Department of Pediatrics, University of Maryland School of Medicine, Baltimore, Maryland, USA; h Institute for Genome Sciences, University of Maryland School of Medicine, Baltimore, Maryland, USA; University of California, San Francisco

**Keywords:** 16S rRNA, absolute abundance, quantitative microbiota analysis, stomach microbiota, transcriptional activity

## Abstract

Clinical interventions in the stomach have been linked to fecal microbiota alterations, suggesting a function of the stomach in gastrointestinal (GI) homeostasis. We sought to determine the taxonomic bacterial biogeography of the upper GI tract, including different sites within the human stomach (cardia, corpus, and antrum), adjacent upstream (esophagus) and downstream (duodenum) locations, and luminal contents (aspirate), as well as whole-stomach samples from mice and gerbils. Qualitative and quantitative DNA- and RNA-based taxonomic microbiota analyses were combined to study the relationship of relative and absolute bacterial abundances and transcriptionally active bacterial microbiota components in the stomach of humans and mice. Stomach microbiota compositions resembled those of esophagus and duodenum. However, along the descending GI tract, the relative abundances of specific oropharyngeal commensals decreased (*Streptococcus*) or increased (Rothia mucilaginosa, *Porphyromonas*, and *Lachnospiraceae*). Furthermore, the compositional similarity (weighted UniFrac) between stomach aspirates and esophageal biopsy samples increased with gastric *Streptococcus* relative abundance. In both human aspirate and mouse stomach samples, *Firmicutes* were more abundant among transcriptionally active bacteria than *Bacteroidetes*. The relative abundance of *Firmicutes* in the stomach was negatively correlated and that of *Bacteroidetes* was positively correlated with absolute bacterial abundance, suggesting a disproportionate increase of *Bacteroidetes* over *Firmicutes* at higher bacterial densities. Human, mouse, and gerbil stomach samples showed similarities at higher taxonomic levels but differences at lower taxonomic levels. Our findings suggest selective enrichment and depletion of specific bacterial taxa in the stomach and *Firmicutes* being transcriptionally more active than *Bacteroidetes* that increase in relative abundance with total bacterial load.

**IMPORTANCE** Clinical stomach interventions, such as acid inhibition or bypass surgery, have been linked to fecal microbiota alterations. We demonstrate that the stomach microbiota largely overlaps those of adjacent gastrointestinal locations and identify gradual decreases and increases in the relative abundances of specific bacteria within the stomach, suggesting selective enrichment and depletion. Moreover, similarities between stomach and esophagus samples are proportional to the concentrations of *Streptococcus* (*Firmicutes*) in the stomach. The relative abundance of *Firmicutes* in the stomach, compared to that of *Bacteroidetes*, is increased in RNA relative to DNA, indicating higher transcriptional activity. Moreover, increased absolute bacterial loads are associated with decreased relative abundance of *Firmicutes* and higher relative abundance of *Bacteroidetes*. Our findings characterize the stomach microbiota as influenced by *Bacteroidetes* influx against a background of transcriptionally more active *Firmicutes.* Human, mouse, and gerbil stomach microbiotas differ at lower taxonomic levels, which might affect the utility of these model organisms.

## Abstract (precis)

Clinical stomach interventions, such as acid inhibition or bypass surgery, have been linked to fecal microbiota alterations. We demonstrate that the stomach microbiota largely overlaps those of adjacent gastrointestinal locations and identify gradual decreases and increases in the relative abundances of specific bacteria within the stomach, suggesting selective enrichment and depletion. Moreover, similarities between stomach and esophagus samples are proportional to the concentrations of *Streptococcus* (*Firmicutes*) in the stomach. The relative abundance of *Firmicutes* in the stomach, compared to that of *Bacteroidetes*, is increased in RNA relative to DNA, indicating higher transcriptional activity. Moreover, increased absolute bacterial loads are associated with decreased relative abundance of *Firmicutes* and higher relative abundance of *Bacteroidetes*. Our findings characterize the stomach microbiota as influenced by *Bacteroidetes* influx against a background of transcriptionally more active *Firmicutes.* Human, mouse, and gerbil stomach microbiotas differ at lower taxonomic levels, which might affect the utility of these model organisms.

## INTRODUCTION

The systemic relevance of the gastrointestinal (GI) microbiota for human health has been widely documented in the scientific literature. Specific microbiota features have been linked to the risk for and recovery from infectious diseases (1–4) and diverse metabolic (5–7) and immunological disorders, including autoimmunity and allergies (8–10), as well as malignant diseases (11, 12) and even neurological conditions (13). Microbiota projects typically focus on fecal samples as a proxy for the gastrointestinal microbiome, although differences in microbial density, diversity, and composition between the small and large intestine and between the luminal and mucosa-adherent microbiota have been reported (14–16). The stomach has received less attention than the intestine, despite the reports from recent studies that linked gastric surgical and medical interventions to alterations of the fecal microbiota and associated clinical features: Roux-en-y gastric bypass surgery altered the lower intestinal microbiota in mice, with beneficial metabolic effects on the host that were transferable by microbiota transplantation to germfree mice (17), and similar effects were shown in humans (18, 19). Suppression of gastric acid production by proton pump inhibitors is associated with increased risk for Clostridium difficile infection (20) and has been demonstrated to alter fecal microbiota compositions in humans (21, 22). An improved understanding of the gastric microbial ecosystem therefore has the potential to generate new means to influence gut homeostasis, with potentially far-reaching systemic health consequences.

The perspective on the stomach microbiota has considerably changed over the last decades. Originally considered sterile due to the harsh acidic and proteolytic environment, the stomach is now assumed to harbor diverse bacterial and fungal communities at the mucosa and in luminal fluid, in addition to the prominent gastric pathogen Helicobacter pylori (23–25). Compositional differences among gastric, oral, and throat microbiota (26) have been interpreted as indicators for the existence of distinct microbial communities that include adapted, residual bacteria in the stomach (23). However, conclusive evidence for a metabolically active unique gastric microbiota is difficult to provide by 16S rRNA gene amplicon sequencing-based microbiota analysis alone.

**FIG 1:**
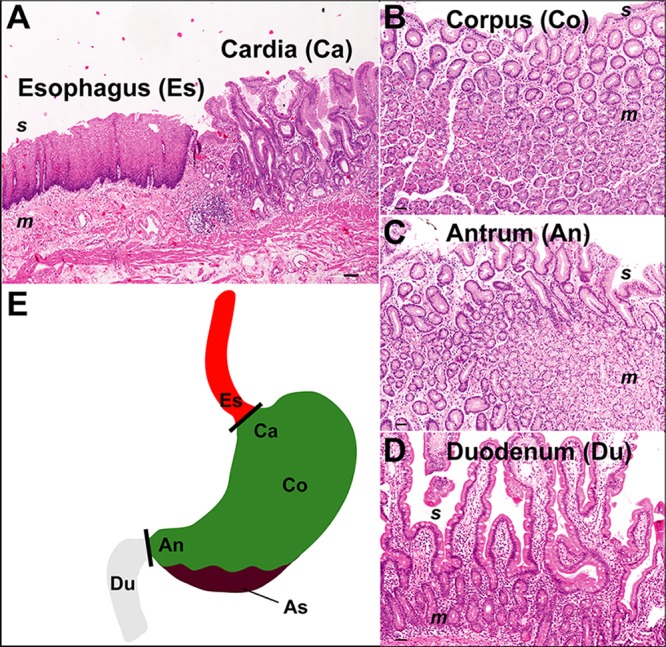
Histology and locations of the sampled human upper GI tract sections. (A) Gastroesophageal junction, showing the stratified multilayered squamous epithelium of the esophagus (Es) and the single layered columnar epithelium of the gastric cardia region (Ca). (B) Stomach corpus (Co) mucosa with oxyntic gastric glands. (C) Stomach antrum (An) with mucous glands in the mucosa. (D) Small-bowel architecture with villi and crypts of the duodenum (Du). (E) Schematic overview, including gastric aspirate (As). Abbreviations: s, surface; m, mucosa. Magnifications, ×40 (A and B) and×100 (C and D).

The stomach differs from adjacent up- and downstream locations of the GI tract with respect to pH (27) and mucosal architecture, and separate sections within the stomach display additional histological variation (Fig. 1). While the esophagus is lined by a stratified multilayered squamous epithelium, a single layer of columnar epithelial cells covers the stomach and duodenum, the latter specifying the beginning of the small intestine. Within the stomach, three different histological regions, cardia, corpus, and antrum, can be distinguished based on the composition of epithelia and glands and on the cellular profiles of secretory compounds. Gastric acid and pepsinogen are produced exclusively in the corpus region via oxyntic glands, whereas specific mucins are produced in cardia and antrum, and all locations vary with respect to the presence of endocrine- and antimicrobial peptide-producing cells (28, 29). Intriguingly, each of the individual stomach regions is affected by a distinct set of diseases, which have been associated with specific microbiota alterations (30–34). Mice and gerbils are two commonly used rodent models of human stomach pathology (35). However, in addition to the glandular region, which is outlined by a simple columnar epithelium and is similar to the human stomach, rodents have a nonglandular forestomach, which provides storage and mechanical food digestion (36). Nevertheless, well-defined boundaries separate the stratified squamous epithelium of the esophagus from both the glandular stomach in humans and the forestomach in rodents (36).

Previous studies on the human stomach microbiota found significant differences between gastric mucosal biopsy samples and fluid aspirate samples (37), with the former being dominated by H. pylori in colonized individuals (38–40) and the latter containing commensal microorganisms from the throat and mouth (41–43). Studies comparing microbiota compositions based on isolated DNA or RNA found parts of the gastric microbiota to display transcriptional activity in the stomach (44), which could indicate resilience or even adaptation of specific bacterial taxa to the harsh environment of the stomach. H. pylori colonization, pH, and immunosuppression were linked to differences in gastric microbiota compositions (44).

Here we sought to generate a high-resolution map of the microbiota of the human stomach, including detailed comparisons of clinically and histologically different gastric locations (cardia, corpus, and antrum) and samples associated with the gastric lining and luminal contents (biopsy samples and aspirates) and adjacent sites that are located up- and downstream of the stomach (esophagus and duodenum). Gastric microbiota compositions were analyzed in the context of two clinically and geographically distinct human cohorts and compared to those of mice and gerbils. To explore the relationship of the total gastric bacterial microbiota that can be identified in the stomach and that may include dead and inactive bacteria and the fraction of the microbiota that retains transcriptional activity, additional 16S rRNA gene and transcript amplicon sequencing was carried out on a small set of human gastric aspirate and mouse whole-stomach samples. Combined qualitative and quantitative bacterial microbiota analyses of these samples were used to compare relative and absolute abundances of specific bacterial phyla.

We demonstrate significant interindividual variations in human and animal gastric microbiota compositions, independent of host disease status, and enrichment and depletion of specific bacterial taxa along the descending axis of the upper human GI tract. Further, 16S rRNA transcript analysis indicates a relatively larger contribution of bacteria from the phylum *Firmicutes* than of bacteria from *Bacteroidetes* to the transcriptionally active stomach microbiota and that interindividual variation in the relative abundances of these two bacterial groups appears to be largely driven by differences in the absolute abundance of *Bacteroidetes* alone.

## RESULTS

### Patient population and sample types.

A diverse patient population was enrolled in this study, including asymptomatic patients and individuals with various GI pathologies, who underwent diagnostic and surveillance upper GI tract endoscopy, respectively. A summary of the clinical patient information is provided in Table 1, and a detailed description of the sample metadata is presented as part of the supplemental material (see Table S1 in the supplemental material). To study the spatial organization of the stomach microbiota, separate topographic locations representative of specific mucosal architectures were sampled (Fig. 1). Specimens consisted of gastric fluid aspirate and biopsy samples from the esophagus, the esophageal-gastric junction (cardia), the gastric corpus and antrum, and the duodenum (small intestine). Specimens were analyzed by 16S rRNA gene amplicon sequencing, which on average generated 3,515 reads corresponding to 98 operational taxonomic units (OTUs) per sample (see Table S2 and Fig. S1 in the supplemental material for overviews of sequence data and taxonomic microbiota composition).

**TABLE 1:** Patient population and analyzed samples^*a*^

Patient	Age (yrs)	Sex	Disease category	Collected samples
P01	68	F	GERD	E, Ca, Co, An, D, As
P02	21	F	IBD (Crohn’s disease)	E, Ca, Co, An, As
P03	61	M	Normal	Ca, Co, An, As
P04	38	F	Normal	E, Ca, Co, An, D, As
P05	23	F	Normal	E, An, D, As
P06	20	F	Normal	E, Co, D, As
P07	29	F	Normal	E, An, D, As
P08	68	M	Atrophic autoimmune gastritis	Ca, Co, An, As
P09	47	F	Normal	E, Ca, An, D
P10	54	F	Normal	E, Co, An, D, As
P11	24	F	Normal	E, Ca, An, D, As
P12	71	F	Normal	E, Ca, Co, An, D, As
P13	61	M	Helicobacter pylori gastritis	E, Ca, Co, As
P14	56	F	Helicobacter pylori gastritis	E, An, As
P15	54	M	Normal	As
P16	27	F	Normal	As
P17	39	F	Atrophic autoimmune gastritis	As

a Abbreviations: F, female; M, male; GERD, gastroesophageal reflux disease; IBD, inflammatory bowel disease; E, esophagus; Ca, cardia; Co, corpus; An, antrum; D, duodenum; As, aspirate. E, Ca, Co, An, and D were collected as biopsy samples. Disease categories are based on histological diagnosis of the stomach. See Table S1 for additional information.

10.1128/mSystems.00262-18.1 FIG S1 Distribution of generated reads and OTUs used for analysis. Numbers of reads and OTUs per samples were compared based on sampled location (A) or on biopsy sample or aspirate (B) and species source (C). Comparisons were carried out for all generated reads (maximum depth) and rarefied data sets (880 reads per sample for human biopsy and aspirate samples and 1,415 reads per sample for human aspirate and gerbil and mouse stomach samples). Significance data were determined based on the following nonparametric tests: the Kruskal-Wallis test with Dunn’s multiple comparison correction (A and C) and the Mann-Whitney U test (B). *, *P* < 0.05; **, *P* < 0.01; ***, *P* < 0.001. Download FIG S1, TIF file, 0.2 MB. Copyright © 2018 Wurm et al. 2018 Wurm et al. This content is distributed under the terms of the Creative Commons Attribution 4.0 International license.

10.1128/mSystems.00262-18.7 TABLE S1 Human clinical patient and animal metadata information. Download Table S1, XLSX file, 0.1 MB. Copyright © 2018 Wurm et al. 2018 Wurm et al. This content is distributed under the terms of the Creative Commons Attribution 4.0 International license.

10.1128/mSystems.00262-18.8 TABLE S2 Overview of generated sequence data and taxonomic microbiota compositions from primary and secondary study (OTU tables). Download Table S2, XLSX file, 1.0 MB. Copyright © 2018 Wurm et al. 2018 Wurm et al. This content is distributed under the terms of the Creative Commons Attribution 4.0 International license.

### Stomach microbiota in relation to esophagus and duodenum.

**FIG 2:**
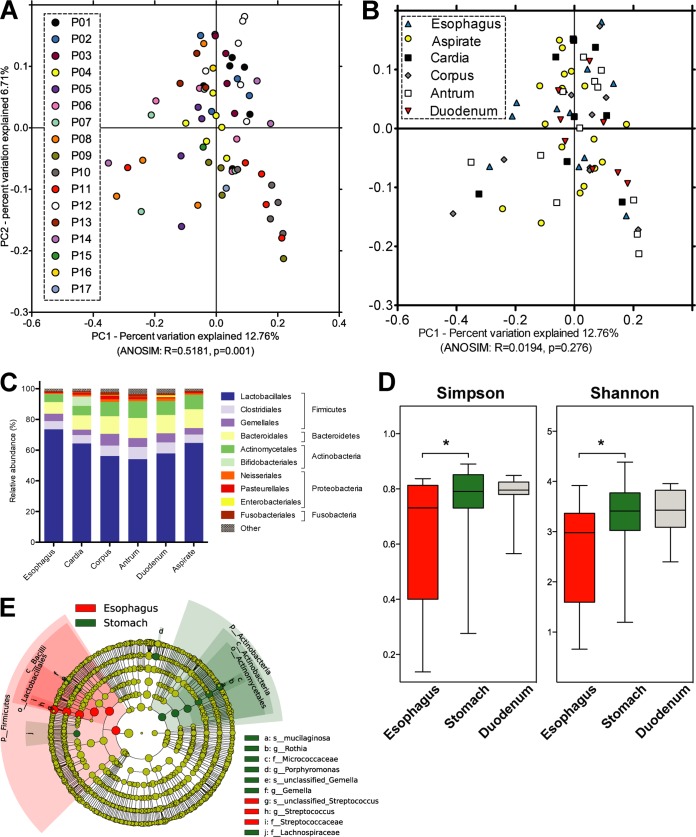
Mucosal microbiota analysis of the human stomach in relation to esophagus and duodenum. (A and B) Principal-coordinate analysis (PCoA) plots of microbiota comparisons of biopsy samples based on taxonomic distance (unweighted UniFrac), showing significant differences between patients (A) but not between sampled locations (B). Significance was determined with a nonparametric analysis of similarity (ANOSIM) test, using 999 permutations, as implemented in QIIME (64). (C) Mean relative microbiota compositions of all upper GI samples at the taxonomic order and phylum levels, including all OTUs with ≥1% relative abundance in at least one sample, excluding samples from H. pylori-positive patients. (D) Cladogram showing bacterial taxa with differential relative abundances, from kingdom (innermost ring) to species (outermost ring), between esophagus and stomach biopsy samples from H. pylori-negative patients, using a linear discriminant analysis (LDA) effect size of >2.0 as determined with LEfSe (65). Circle diameters used to represent taxa are proportional to their relative abundances. Circle colors indicate a significant increase in esophagus samples (red) or in stomach samples (green) or no difference between the two sample types. Shaded circle fractions in red and green mark the lower taxonomic groups that comprise each taxon with significantly different relative abundances in the two sample groups.

Based on phylogenetic (unweighted/weighted UniFrac) and other (Bray-Curtis) distance metrics, stomach, esophagus, and duodenum samples clustered by individual (*P* = 0.001) rather than by specimen location (*P* > 0.1) (Fig. 2A and B). For all locations and specimen types, microbiota compositions were dominated by the phylum *Firmicutes* (73.2 ± 1.2%), followed by *Bacteroidetes* (12.5 ± 0.9%), *Actinobacteria* (9.4 ± 0.6%), and *Proteobacteria* (2.9 ± 0.4%) (Fig. 2C), except in two patients with H. pylori gastritis (P13 and P14), as diagnosed histologically and by immunostaining. For these H. pylori-positive patients, in agreement with the preferred mucosal niche of this stomach-adapted bacterium, gastric biopsy samples but not aspirate or esophageal biopsy samples contained high relative abundances of H. pylori, with <0.5% (esophagus), >95% (corpus and antrum), >22% (cardia), and <5% (gastric aspirate) of all reads assigned to this bacterium (Fig. S2; the duodenum was not sampled from these individuals). In addition to those patients, 20 of 60 samples from 15 patients not diagnosed as H. pylori positive contained H. pylori at relative abundances of 0.016% to 3.632%, suggesting low-level colonization in these individuals.

10.1128/mSystems.00262-18.2 FIG S2 Mean relative microbiota compositions in H. pylori-positive samples. The histogram shows the taxonomic classifications at the genus and phylum levels of all OTUs with ≥1% relative abundance in at least one sample. Download FIG S2, TIF file, 0.5 MB. Copyright © 2018 Wurm et al. 2018 Wurm et al. This content is distributed under the terms of the Creative Commons Attribution 4.0 International license.

**FIG 3:**
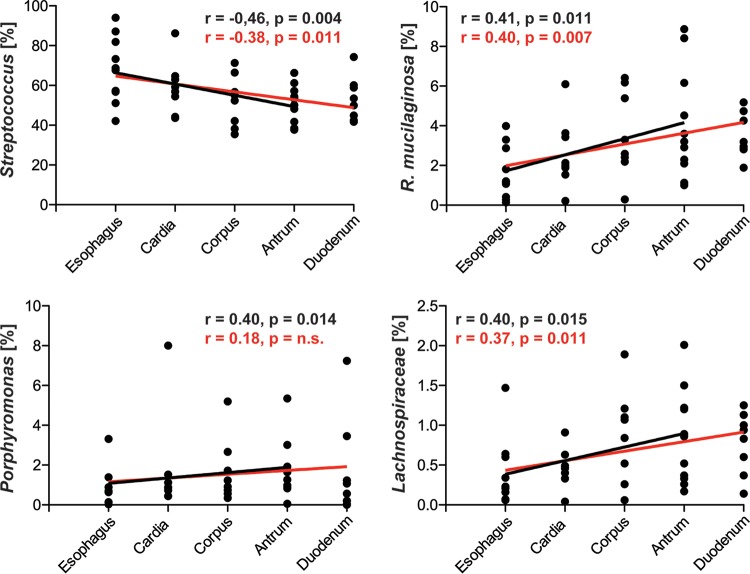
Changes in relative abundance of specific bacterial taxa along the descending axis of the human upper GI tract. Relative abundances of bacterial taxa with a mean relative abundance of >0.1% and an LDA effect size of >2.0 for the LEfSe comparison of stomach and esophagus biopsy samples from H. pylori-negative patients (Fig. 2D) were analyzed. Correlations were determined separately from esophagus to antrum (black lines) and from esophagus to duodenum (red lines), using the two-tailed, nonparametric Spearman’s rank correlation coefficient test.

A comparison of the individual stomach locations showed neither significant differences in overall taxonomic microbiota composition (without samples from H. pylori-positive patients; Fig. 2A and B) nor significant differences in microbial diversity (Shannon/Simpson indices, excluding samples from H. pylori-positive patients; Fig. S3A and B). While microbial diversity tended to be increased in gastric compared to esophageal biopsy samples from H. pylori-negative patients (*P* < 0.05, based on Shannon/Simpson indices [not significant for rarefaction analysis]) but not compared to gastric and duodenal biopsy samples (Fig. S3C and D), four bacterial groups with a mean relative abundance of >0.1% showed significant differences between stomach and esophageal biopsy samples (linear discriminant analysis [LDA] score of >2.0) (Fig. 2D). Mean relative abundances of the genus *Streptococcus* were decreased (68.0 ± 5.2% versus 53.8 ± 2.3%) (LDA = 4.9, *P* = 0.014) and those of the species Rothia mucilaginosa (1.62 ± 0.42% versus 3.48 ± 0.44%) (LDA = 4.0, *P* = 0.018), the genus *Porphyromonas* (0.86 ± 0.3% versus 1.73 ± 0.35%) (LDA = 3.7, *P* = 0.034), and the family *Lachnospiraceae* (0.39 ± 0.14% versus 0.74 ± 0.1%) (LDA = 3.3, *P* = 0.037) were increased in stomach compared to esophagus biopsy samples. R. mucilaginosa abundances were also increased in duodenum compared to esophagus biopsy samples, whereas no differences were detected between stomach and duodenum samples. Those bacterial taxa with different overall stomach and esophagus relative abundances also displayed gradual changes within the stomach (Fig. 3). The relative abundance of *Streptococcus* continually decreased (Spearman’s rank correlation coefficient, *R* = −0.46, *P* < 0.005) along the GI axis, i.e., from esophagus to cardia, corpus, and antrum, whereas those of R. mucilaginosa, *Porphyromonas*, and *Lachnospiraceae* continually increased (*R* > 0.39, *P* < 0.02). If duodenal biopsy samples were included in the analysis, the strength of correlation between increasing or decreasing relative abundances and progression along the GI tract would be reduced for all four taxa (Fig. 3).

10.1128/mSystems.00262-18.3 FIG S3 Alpha diversity comparisons of microbiotas of different samples types within the stomach and between esophageal, gastric, and duodenal mucosal biopsy samples. Samples from H. pylori-negative patients were compared based on Shannon (A and C) and Simpson (B and C) diversity indices and rarefaction analysis (D), as implemented in QIIME (37). Analyses were run on data sets rarefied to 880 reads per sample, and significance data were calculated based on nonparametric two-sample *t* tests, using 999 Monte Carlo permutations and Bonferroni correction for multiple comparisons (*, *P* < 0.05). Download FIG S3, TIF file, 1.4 MB. Copyright © 2018 Wurm et al. 2018 Wurm et al. This content is distributed under the terms of the Creative Commons Attribution 4.0 International license.

**FIG 4:**
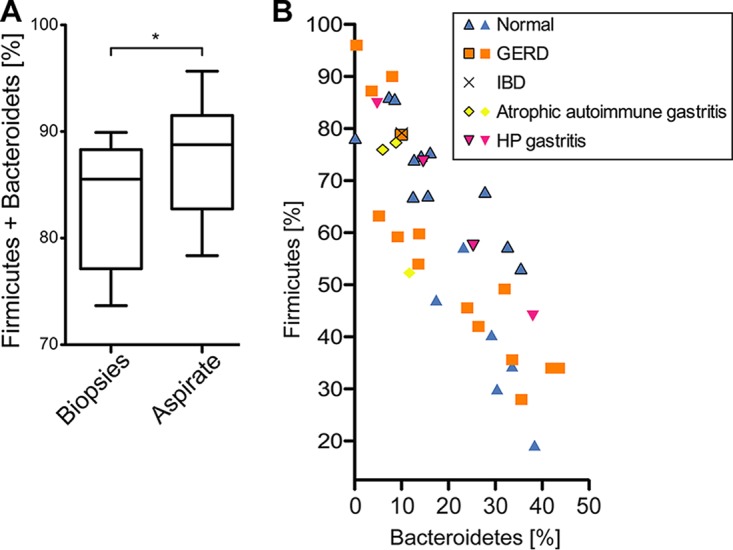
*Firmicutes* and *Bacteroidetes* in human gastric aspirate samples. (A) The relative dominance of *Firmicutes* and *Bacteroidetes* over the gastric microbiota was more pronounced in gastric aspirate samples than in biopsy samples, as demonstrated by the results of comparisons of combined fractions performed using the nonparametric Mann-Whitney *U* test (*, *P* < 0.05). (B) Variations in the relative abundances of *Firmicutes* and *Bacteroidetes* among gastric aspirate samples are apparently not linked to patient disease status. Symbols represent samples from individuals with different disease backgrounds from two separate studies, i.e., data from this study (outlined symbols) and a previously published data set (44), including H. pylori (HP)-positive and-negative patients.

The human gastric aspirate samples exhibited levels of microbial diversity (Fig. S3A and B) and taxonomic compositions (Fig. 2C) similar to those seen with the biopsy samples, but the dominance of *Firmicutes* and *Bacteroidetes* over the entire microbiota was even more pronounced for gastric fluid samples (mean, 87.7 ± 1.4%) than for biopsy samples (mean, 83.5 ± 1.1%) (Fig. 4A). Relative abundance ranges differed substantially between individuals (for *Firmicutes*, 53.1% to 86.1%; for *Bacteroidetes*, 0.2% to 35.5%) but were not significantly linked to patient health status (Fig. 4B). Reanalysis of a previously published gastric aspirate data set from a heterogeneous patient cohort (44) showed similar degrees of variation irrespective of patient health status (Fig. 4B; *P* > 0.1 for all data combined [Kruskal-Wallis rank sum test]).

### Relationship of stomach and adjacent microbiota.

**FIG 5:**
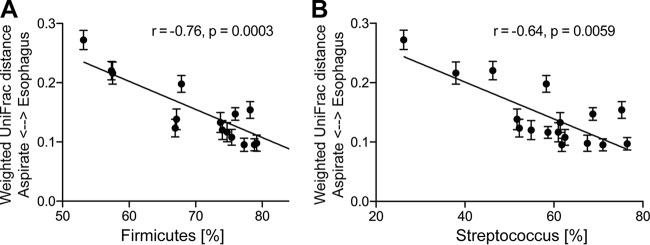
Relationship of human stomach and esophagus microbiota. Analyses of correlations of taxonomic distances (weighted UniFrac) between gastric aspirate and esophageal biopsy samples with the relative abundances of the phylum *Firmicutes* (A) or the genus *Streptococcus* (B) in aspirate samples were performed on the basis of Spearman’s (two-tailed) rank correlation coefficient analysis. Each bar shows the relative abundance of *Firmicutes* (A) or *Streptococcus* (B) of an individual aspirate sample, as well as the range of taxonomic distances found between the microbiota of this sample and all esophagus biopsy samples, including samples from H. pylori-positive and -negative patients.

To study the relationship between the microbiota of esophagus and stomach, pairs of human esophageal biopsy and gastric fluid samples were compared and the similarities in microbiota composition calculated based on phylogenetic distance (weighted UniFrac). Gastric aspirate samples were selected for this analysis, as the aspirate microbiota was expected to be more strongly influenced by microbial influx from upstream GI locations than the mucosal microbiota from biopsy samples. Phylogenetic distances between gastric fluid and esophagus microbiota pairs were negatively correlated with the relative abundance of the most abundant member of the gastric microbiota, i.e., the phylum *Firmicutes* (Spearman *R* = −0.76, *P* = 0.0003) or the genus *Streptococcus* (Spearman *R* = −0.64, *P* = 0.006) (Fig. 5). In other words, a higher relative abundance of *Firmicutes* or *Streptococcus* in gastric fluid was associated with a higher level of microbiota composition similarity of gastric fluid to esophageal biopsy samples.

### Comparison of human, mouse, and gerbil gastric microbiotas.

**FIG 6:**
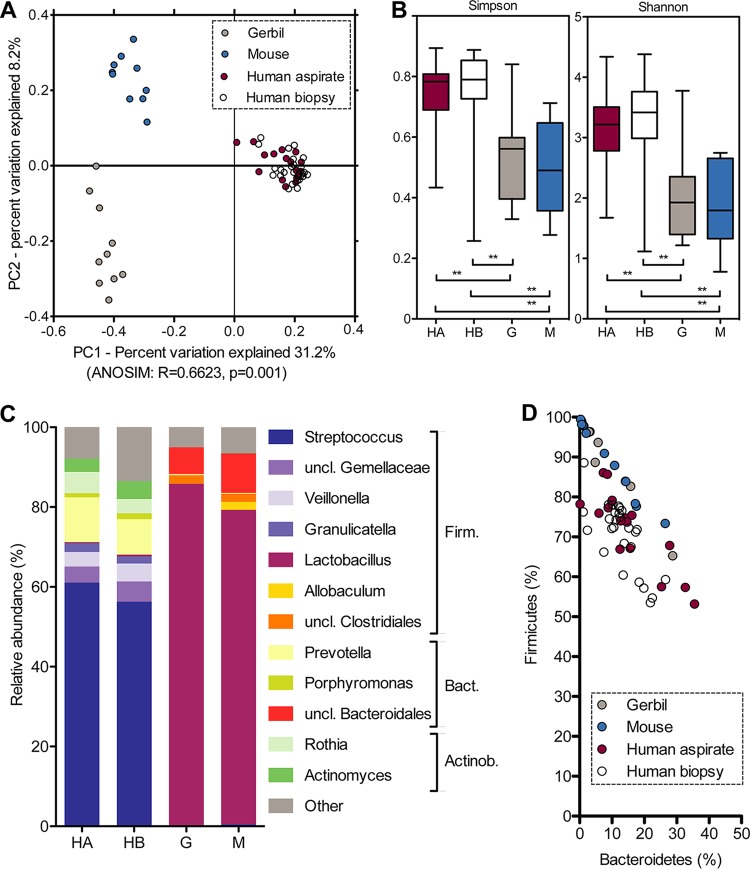
Microbiota comparison of H. pylori-negative human, mouse, and gerbil stomach samples. (A) Taxonomic microbiota compositions differ between humans (aspirate plus biopsy samples), mice, and gerbils, as determined with nonparametric ANOSIM tests, using 999 permutations, based on taxonomic distance (unweighted UniFrac). (B) Microbial diversity comparison using Shannon and Simpson indices, with significance determined based on a nonparametric two-sample *t* test, using 999 Monte Carlo permutations and Bonferroni correction for multiple comparisons (**, *P* < 0.01). (C) Mean relative microbiota compositions at the genus and phylum levels, including all OTUs with ≥1% relative abundance in at least one group of samples (human, mouse, or gerbil). (D) Variations in the relative abundances of *Firmicutes* (Firm.) and *Bacteroidetes* (Bact.) among human, mouse, and gerbil samples. Actino., *Actinobacteria*; HA, human aspirate; HB, human biopsy; G, gerbil; M, mouse; uncl., unclassified.

To compare the human stomach microbiota to that of two animal models for gastric disease, whole-stomach samples from healthy adult individually housed or cohoused mice and gerbils were included in the analysis (see Table S1 for animal background). Gastric samples clustered by host (unweighted/weighted UniFrac, Bray-Curtis distance, *P* = 0.001) (Fig. 6A; see also Fig. S4A and B) and showed increased microbial diversity in humans compared to animals (Simpson/Shannon index; Fig. 6B; not significant for rarefaction analysis, Fig. S4C). Human and animal samples were dominated by the same two bacterial phyla, classes, and orders (*Firmicutes*, *Bacilli*, *Lactobacillales* and *Bacteroidetes*, *Bacteroidia*, *Bacteroidales*) (Fig. 6C). However, human samples mostly contained *Streptococcus* (59.5 ± 13.5%) and *Prevotella* (12.9 ± 2.2%), whereas mouse and gerbil samples were dominated by *Lactobacillus* (81.9 ± 13.8%) and an unknown genus of the family *Muribaculaceae* (formerly S24-7; 8.5 ± 1.9%). Animal samples showed interindividual variations in the relative abundances of *Firmicutes* and *Bacteroidetes* similar to those seen in human samples (Fig. 6D). While the animal sample numbers were too low for statistical analysis, no obvious link was apparent between *Firmicutes* and *Bacteroidetes* relative abundance and animal age, sex, genetic relationship, or caging condition (Table S1).

10.1128/mSystems.00262-18.4 FIG S4 Microbiota composition of human aspirate and mouse and gerbil whole-stomach samples. Comparisons of samples were performed on the basis of taxonomic distance using weighted UniFrac (A) and Bray-Curtis dissimilarity (B) data and on the basis of microbial diversity using rarefaction analysis (C), as implemented in QIIME (37). Analyses were run on data sets rarefied to 1,415 reads per sample, and significance was determined with the nonparametric analysis of similarity (ANOSIM) test, using 999 permutations (A and B), or with nonparametric two-sample *t* tests, using 999 Monte Carlo permutations and Bonferroni correction for multiple comparisons (C). Human samples were from H. pylori-positive and -negative patients. Download FIG S4, TIF file, 1.0 MB. Copyright © 2018 Wurm et al. 2018 Wurm et al. This content is distributed under the terms of the Creative Commons Attribution 4.0 International license.

### 16S rRNA gene- and transcript-based quantitative stomach microbiota analysis.

An additional set of seven human gastric aspirate and six mouse whole-stomach samples was analyzed by 16S rRNA gene and transcript amplicon sequencing and quantitative 16S rRNA gene PCR (Table S1). This secondary study was conceived to demonstrate the feasibility of a quantitative assessment of the total and transcriptionally active bacterial fraction within the stomach microbiota and to support findings from the primary data set.

Metagenomic DNA and metatranscriptomic RNA were simultaneously isolated from specific and comparable sample quantities. Exactly the same bacterial lysis protocol was applied for DNA and RNA isolation, and two technical replicates were processed per sample (Fig. S5). Relative taxonomic microbiota compositions were determined by 16S rRNA gene or transcript amplicon sequencing to a minimum depth of 10,000 reads per sample (Table S2). The yields of extracted DNA and RNA and of quantitative 16S rRNA gene copies and transcripts, as determined by 16S rRNA-specific PCR or reverse transcription-PCR (RT-PCR), were normalized to data from 1 ml of gastric aspirate, in order to compare analysis results from different human individuals. For mice, nucleic acid yields and 16S rRNA gene copy and transcript numbers were normalized to data from one complete mouse stomach (see Materials and Methods for details). Among all parameters, mean values from two replicates were used for subsequent analyses.

10.1128/mSystems.00262-18.5 FIG S5 Comparisons of technical replicates based on taxonomic bacterial microbiota compositions. Bar plots show similarities in taxonomic bacterial microbiota composition (beta-diversity), based on Bray-Curtis dissimilarity, between technical replicates and nonreplicates. Similarities are compared for all human gastric aspirate and mouse whole-stomach sample data combined (gray bar) and for human and mouse DNA-based data (blue bar) and RNA-based data (orange bar) separately (***, *P* < 0.001 [Wilcoxon signed rank text with Bonferroni correction]). Download FIG S5, TIF file, 0.5 MB. Copyright © 2018 Wurm et al. 2018 Wurm et al. This content is distributed under the terms of the Creative Commons Attribution 4.0 International license.

On average, human gastric aspirate contained 324 ng DNA (± 76.2), including 7 × 10^8^ 16S rRNA gene copies (± 6.6 × 10^8^), and 429 ng RNA (± 143.2), including 3 × 10^7^ 16S rRNA transcripts (± 1.9 × 10^7^), per ml of aspirate, compared to 4.5 μg DNA (± 1.08), including 3 × 10^9^ 16S rRNA gene copies (± 1.1 × 10^9^), and 13.0 μg RNA (± 4.39), including 4 × 10^8^ 16S rRNA transcripts (± 2.2 × 10^8^), per mouse stomach. Total yields of DNA and RNA, as well as of 16S rRNA gene copies and transcripts per milliliter of human gastric aspirate and mouse whole-stomach samples, were positively correlated, and so were DNA yield and 16S rRNA gene copies (all *P* < 0.05 [Spearman’s rank correlation]) but not RNA yield and number of 16S rRNA transcripts (*P* > 0.05; Fig. S6). Human and mouse samples contained on average 9.3-fold more 16S rRNA gene copies than transcripts.

10.1128/mSystems.00262-18.6 FIG S6 Correlation analyses of DNA and RNA yields and 16S rRNA gene copy and transcript numbers. DNA and RNA yield data represent nanograms per milliliter of human gastric aspirate (H) or mouse whole-stomach (M) samples. 16S rRNA gene copy numbers and levels of transcripts are presented relative to absolute copy numbers per milliliter of human gastric aspirate or mouse whole-stomach samples. Correlation coefficients (*R*) and significance (*P*) were calculated using Spearman’s rank correlation. Download FIG S6, TIF file, 0.2 MB. Copyright © 2018 Wurm et al. 2018 Wurm et al. This content is distributed under the terms of the Creative Commons Attribution 4.0 International license.

**FIG 7:**
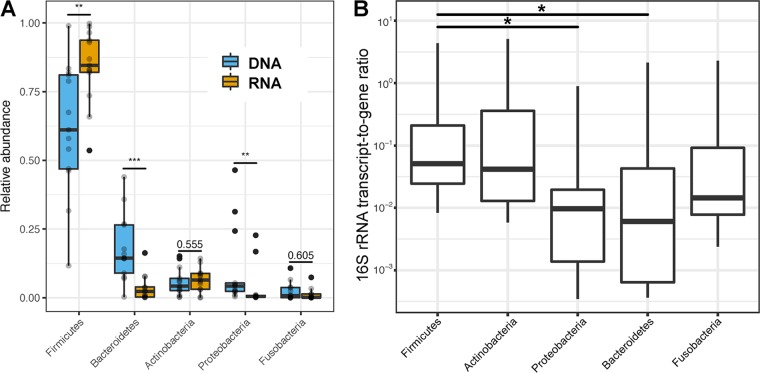
DNA- and RNA-based relative and quantitative taxonomic microbiota compositions of human gastric aspirate (*n* = 7) and mouse stomach (*n* = 6) samples. (A) Comparison of phylum relative abundances in DNA (blue) and RNA (orange) extracts of all samples based on 16S rRNA gene PCR and transcript RT-PCR amplicon sequencing (***, *P* < 0.001; **, *P* < 0.01 [Mann-Whitney U test]). (B) Comparison of bacterial phyla based on the ratio of 16S rRNA transcripts to 16S rRNA genes per sample (*, *P* < 0.05 [Kruskal-Wallis test]).

In agreement with the primary data set, *Firmicutes* and *Bacteroidetes* represented the two most abundant bacterial taxa in DNA and RNA extracts from both human and mouse stomach samples (Table S2). However, in contrast to previously reported findings from our group (44), the direct comparison of DNA- and RNA-based taxonomic microbiota compositions in all human and mouse samples revealed a higher relative abundance of *Firmicutes* and *Actinobacteria* and a reduced relative abundance of *Bacteroidetes* and *Proteobacteria* among 16S rRNA transcripts compared to 16S rRNA gene copies (Fig. 7A). As an additional indicator of transcriptional activity, we also calculated the ratio of absolute 16S rRNA transcript numbers to gene copy numbers (Fig. 7B). This ratio differed significantly between bacterial phyla, ranging from 0.51 ± 0.34 16S rRNA transcripts per gene copy in *Firmicutes* to 0.18 ± 0.16 16S rRNA transcripts per gene copy in *Bacteroidetes*.

**FIG 8:**
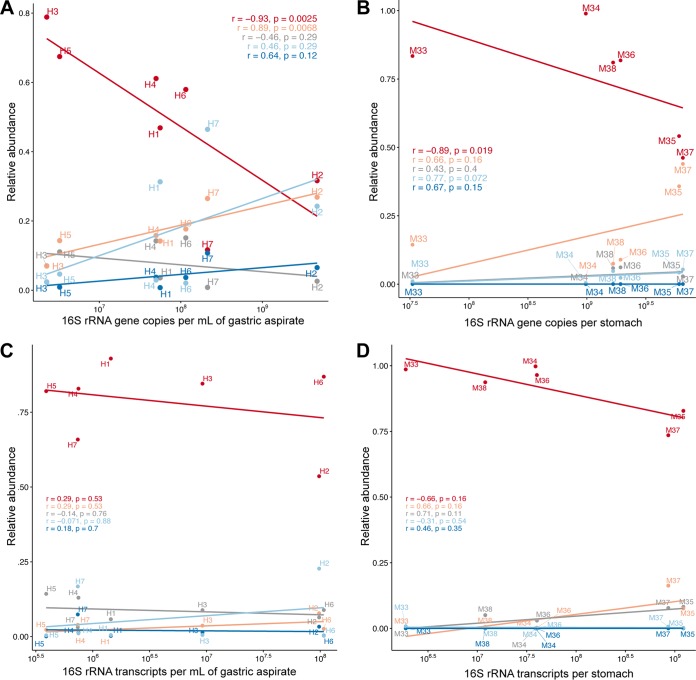
Association of phylum relative abundance and absolute 16S rRNA gene and transcript copy number. Correlations are shown for the five most abundant phyla, including *Firmicutes* (red), *Bacteroidetes* (orange), *Actinobacteria* (gray), *Proteobacteria* (light blue), and *Fusobacteria* (dark blue*).* (A and B) Correlation of phylum relative abundance in DNA, based on 16S rRNA gene amplicon sequencing with bacterial 16S rRNA gene copy number in human gastric aspirate (A) and mouse whole-stomach (B) samples. (C and D) Correlation of phylum relative abundance in RNA, based on 16S rRNA transcript amplicon sequencing and bacterial 16S rRNA transcript number in human gastric aspirate (C) and mouse whole-stomach (D) samples. Correlation coefficients (*R*) and significance (*P*) were calculated using Spearman’s rank correlation.

Interestingly, a higher total bacterial abundance, based on 16S rRNA gene copy number, in gastric human and mouse samples was associated with decreased relative abundance of *Firmicutes* (*P* < 0.02 [Spearman’s rank correlation]) and increased relative abundance of *Bacteroidetes* (*P* < 0.01; not significant for mouse samples) (Fig. 8A and B). However, no correlation was observed between 16S rRNA transcript numbers and phylum relative abundances in RNA isolates (*P* > 0.05; Fig. 8C and D), indicating that absolute bacterial densities in human gastric aspirate and mouse whole-stomach samples have a significant impact on the relative taxonomic composition of the total (i.e., DNA-based) gastric microbiota but not the transcriptionally active (i.e., RNA-based) gastric microbiota.

## DISCUSSION

We identified a stomach-specific microbiota of increased microbial diversity and altered taxonomic composition compared to esophagus but not duodenum. Within the stomach, our analysis of the mucosal microbiota showed marginal compositional variation between cardia, corpus, and antrum, despite well-known differences in histological organization and pathological relevance between these sites (45). However, increases and decreases in the relative abundances of specific bacteria in gastric biopsy samples correlated with the distance from the esophagus along the GI axis, suggesting that while the microbiota of the stomach is closely linked to the esophagus, it is also gradually modulated during gastric passage. Interestingly, opposite effects were observed for different bacterial taxa that are typically described as oral commensals (46, 47), including selective depletion of *Streptococcus* and enrichment of R. mucilaginosa and *Porphyromonas* in the stomach, which highlights the need to differentiate among specific microbial taxa when describing the effects of stomach passage on ingested bacteria.

In our study, the observed microbiota modulation from esophagus to stomach was attenuated or even reversed between stomach and duodenum, indicating that the stomach might serve as a bottleneck for bacterial transit from the upper to the lower GI tract. These findings are supported by a recent comparison of metabolically active microbial communities from the upper GI tract, which demonstrated increased relative abundances of *Porphyromonas* and *Lachnospiraceae* as well as decreased relative abundances of *Streptococcus* in stomach compared to duodenal aspirates (48). In addition to differences in histological architecture, physiological factors such as reduced acidity in the duodenum compared to in the stomach (pH ∼6 versus pH ∼1) (27) or the presence of bile acids (49) could exert different effects on mucosal microbial communities. We have previously demonstrated that pH and immunosuppression correlate with alterations in gastric microbiota compositions (44), in line with studies linking use of proton pump inhibitors to increased risk for C. difficile infection (20). Microbiota shifts between stomach and duodenum support the idea of a gastric role for controlling access of ingested bacteria to the lower GI tract, which could reduce the risk of pathogen infection (23) and result in oral strains surviving transit to the gut only in small quantities (50). Intestinal colonization with specific opportunistic bacteria from the salivary microbiota (e.g., *Klebsiella* spp.) can induce intestinal inflammation and has been associated with inflammatory bowel diseases (51). Interference with the entire stomach microbiome could therefore be one factor to explain previous reports of altered lower GI tract microbiota compositions as a consequence of bariatric surgery and gastric acid inhibition and could play a role with respect to the widely used but poorly defined concept of dysbiosis (52).

Previous studies have examined the extent to which the stomach harbors a specific, residual microbiota that is distinct from those of adjacent sites. Oral commensal bacteria are frequently identified in the stomach, and the stomach microbiota overlaps those of the oro- and nasopharynx (40–43). A recent 16S rRNA transcript-based microbiota survey suggested saliva as the source of bacteria in gastric aspirate and biopsy samples, specific taxa of which were enriched in the stomach (48). Franzosa et al. identified specific bacterial species (e.g., Streptococcus salivarius) in analyses of the salivary microbiota and fecal metagenomic DNA and metatranscriptomic RNA from the same individual, indicating that even live bacteria could transit from mouth to colon and maintain transcriptional activity (50). Our findings of gradual microbiota changes between esophagus and stomach and within the stomach support the model of a continuous microbial ecosystem of the GI tract and emphasize the need for additional system-wide studies of the GI microbiota.

As a proof of concept, we used a combination of qualitative and quantitative DNA- and RNA-based taxonomic microbiota analyses to study the relationship of relative and absolute bacterial densities and transcriptionally active bacterial microbiota components in the stomach, which could be exemplary for other human microbiota studies, particularly those addressing samples of low microbial biomass and unclear metabolic activity. Consistent with previous studies on the stomach (30, 41, 43, 48, 53), we demonstrated large interindividual variations in the relative abundances of the two dominant taxa in the human and animal stomach (*Firmicutes* and *Bacteroidetes*). The ratio of *Streptococcus* (*Firmicutes*) to *Prevotella* (*Bacteroidetes*) in the stomach mucosa (corpus and antrum) has previously been negatively correlated with hiatal hernia length (30), and reduced relative abundance of *Firmicutes* in esophageal biopsy samples was previously shown to be associated with esophagitis and Barrett’s esophagus (54). However, we found no association of either of the two bacterial groups with disease status in human patients or other human, mouse, or gerbil background parameters. In contrast, we provide evidence for differences in absolute bacterial loads being at least partially responsible for the observed variations in relative abundance, with gastric relative abundances of *Bacteroidetes* but not *Firmicutes* being positively correlated with total bacterial density in the stomach. Extreme interindividual variation in the relative abundances of *Firmicutes* and *Bacteroidetes* from the fecal microbiota of healthy human individuals was also reported from the U.S. Human Microbiome Project (55), suggesting that similar non-disease-associated, intrinsic factors could also play a role in shaping the intestinal microbiota.

The physiological roles of *Firmicutes* and *Bacteroidetes* in the stomach could be further differentiated based on the comparison of DNA- and RNA-based relative bacterial microbiota compositions. Using the 16S rRNA transcript-to-gene ratio as a marker of transcriptional activity, *Bacteroidetes* and *Firmicutes* represent the lowest (0.18) and highest (0.51) values, respectively, among the gastric phyla, suggesting increased metabolic activity of *Firmicutes* compared to *Bacteroidetes*. It should be noted that, on the basis of comparisons to Escherichia coli, which has been estimated to contain 6,800 ribosomes and 12.4 rRNA genes per cell under conditions of growth at a doubling time of 100 min (56), which corresponds to a 16S rRNA transcript-to-gene ratio of ∼5 × 10^2^, all gastric bacteria exhibit low transcriptional activity. While this could attest to the harsh gastric conditions that might generally inhibit bacterial transcriptional activity, the possibility cannot be ruled out that experimental bias led to reduced yields of RNA compared to DNA. Together, our findings from the comparative analyses of relative and absolute bacterial abundances and DNA- and RNA-based relative compositions support a model of the stomach microbiota as being dominated by transcriptionally more active *Firmicutes* but also as variably affected by additional, temporal influx of transcriptionally less active *Bacteroidetes*.

We found the overall microbiota similarity between stomach and esophagus, measured as taxonomic distance, to be positively correlated with gastric relative abundances of *Firmicutes*. Influx of *Bacteroidetes* would therefore have to originate from naso- or oropharyngeal locations upstream of the esophagus or from the lower GI tract. Bassis et al. identified the highest relative abundance of the *Bacteroidetes* genus *Prevotella* in human oral wash samples compared to nasal swabs and bronchoalveolar lavage fluid and gastric aspirate samples (41). While those authors, in contrast to our study, found no evidence of selective elimination of *Prevotella* in the stomach, this analysis was performed on 16S rRNA gene-based relative abundance data. Longitudinal sampling of the gastric and naso- and oropharyngeal microbiota in healthy individuals will be needed to test for intraindividual temporal variations and interdependencies of the stomach and esophagus microbiota and to identify the potential source of *Bacteroidetes* in the stomach.

Mice and gerbils are two widely used models to study human stomach pathologies (57). Our gastric microbiota comparisons revealed that humans, mice, and gerbils are dominated by the same two bacterial phyla, classes, and orders. However, the stomach microbiotas of the two rodent species differ from those of humans at the taxonomic family and genus level, which, in light of phenotypic differences in colonization resistance and pathology of mice and gerbils infected with human isolates of H. pylori (58), highlights the need for further studies on the functional relevance of these taxonomic differences.

In conclusion, despite the limitations of the present study, including relatively small sample sizes, we demonstrated the feasibility of combined qualitative and quantitative DNA- and RNA-based microbiota analyses for the stomach, which we found (i) to be closely related to microbial communities from upstream GI locations, (ii) to selectively enrich and deplete specific bacterial taxa during their passage to the duodenum, and (iii) to be shaped by transcriptionally more active *Firmicutes* and less active *Bacteroidetes* that increase in relative abundance with total bacterial stomach load.

## MATERIALS AND METHODS

### Human study cohort and sample collection.

Gastrointestinal biopsy samples and aspirate samples were collected at the Medical University of Graz (Graz, Austria) from 24 individuals who underwent upper endoscopy for various clinical indications (Table 1; see also Table S1 in the supplemental material). The Institutional Review Board of the Medical University of Graz approved the study under protocol number EK 23-212 ex 10/11, and all subjects provided written informed consent to participate in the study. Endoscopic and histologic reports indicated no or minimal evidence of GI pathology in 15 patients and gastritis in 8 patients (H. pylori-associated gastritis, 2 patients; atrophic autoimmune gastritis, 2 patients; other gastritis, 4 patients). One patient had Crohn’s disease. Biopsy samples were obtained by routine esophagogastroduodenoscopy by a gastroenterologist (C. Högenauer). The samples were taken in the following order: (i) duodenum, (ii) gastric fluid, (iii) antrum, (iv) corpus, (v) cardia, (vi) esophagus. Biopsy samples were immediately transferred to RNAlater (Qiagen), and gastric aspirate samples for qualitative microbiota analysis were diluted at a ratio of 1:1 in 0.5 ml RNAlater and stored at −80°C. Aspirate samples for quantitative microbiota analysis were stored on ice for 30 min and centrifuged for 15 min at 13,200 rpm (4°C), and the resulting pellets were diluted with 400 to 800 µl RNAlater. Samples from the primary patient cohort were shipped on dry ice to the Institute for Genome Sciences of the University of Maryland School of Medicine (Baltimore, MD) for processing and sequencing. Gastric aspirate samples from the seven patients who participated in the proof-of-concept study were shipped on dry ice to the University of Hohenheim, Stuttgart, Germany. All samples were stored at −80°C until processing.

### Animal sample collection.

C57BL/6 mice were ordered from the Jackson Laboratory, Bar Harbor, ME, and used to extract stomach samples within 5 days after delivery at the University of Maryland, Baltimore. Mongolian gerbil stomach samples were collected at the Max von Pettenkofer-Institute in Munich, Germany. Samples from 10 mice and 10 gerbils were collected as follows. The animals were euthanized, and complete stomachs, including the forestomach, were extracted, cut in half at the *curvatura gastrica major*, and immediately transferred to RNAlater. Gerbil samples were shipped at room temperature to the University of Maryland, Baltimore. All samples were stored at −80°C upon arrival at the Institute for Genome Sciences, University of Maryland, Baltimore, until processing. For the proof-of-concept study, whole-stomach samples from six C57B46J mice from the animal care facility at the University of Hohenheim were harvested and stored as described above. However, loose stomach content was removed and the remaining stomach tissue cut into two halves, which were used as technical replicates for downstream processing and analyses.

### DNA and RNA extraction.

Metagenomic DNA was isolated from all samples using a previously described protocol (44), which includes both enzymatic digestions (lysozyme, mutanolysin, lysostaphin, proteinase K, and RNase) and mechanical disruption by bead beating. Briefly, stomach and biopsy samples were subjected to vortex mixing at full speed for 3 min, and the entirety of the fluid was transferred to new tubes. Samples were centrifuged at 13,200 rpm for 10 min, the supernatant was discarded, and the pellets were resuspended in phosphate-buffered saline (PBS) for enzymatic and mechanical lysis. Hypervariable regions V1 to V3 of the bacterial 16S rRNA gene were amplified using barcoded primers 27F and 534R with Roche/454 adaptors and purified amplicon mixtures sequenced at the Institute for Genome Sciences, University of Maryland, using 454 primer A and protocols recommended by the manufacturer (Roche), as described previously (59).

For the proof-of-concept study, DNA and RNA were extracted simultaneously by the use of a ZymoBIOMICS DNA/RNA Miniprep kit (Zymo Research). Human aspirate and mouse samples were subjected to vortex mixing for 5 min at 4°C. Two aliquots of 2 to 4 ml of gastric aspirate were used as technical replicates for extraction and centrifuged at 15,000 × *g* for 10 min at 4°C. The luminal side of the mouse stomach tissue was rinsed with 1 ml PBS, and the fluid was centrifuged at 15,000 × *g* for 10 min at 4°C All pellets were washed twice with PBS, resolved in 800 µl of ZymoBIOMICS DNA/RNA lysis buffer, transferred to MP-Lysing matrix tube B (MP Biomedicals), and stored on ice for 5 min. Samples were lysed mechanically by bead beating at 6 m/s for 45 s twice, with intermediate storage on ice for 5 min. The lysate was used as input for the ZymoBIOMICS DNA/RNA Miniprep kit following the manufacture’s recommendations with modifications as follows. The RNA fraction of the samples was treated with DNase I at 37°C for 30 min; DNA and RNA were washed twice with 400 µl wash buffer before elution in 30 to 35 µl DNase/RNase-free water. DNA was stored at −20°C and RNA at −80°C until further processing. Potential additional DNA contamination in RNA isolates was further digested by the use of a Turbo DNA-free kit (Thermo Fisher Scientific).

### 16S rRNA gene and transcript amplification and sequencing.

RNA was reverse transcribed with primer 1492R (l) primer, using a GoScript reverse transcriptase kit (Promega). In brief, 3.5 µl of the DNA-free RNA was incubated with 1.5 µl primer (15 pmol) for 5 min at 70°C and immediately chilled in ice water for 5 min. A reverse transcription reaction mixture, containing 4 µl GoScript 5× reaction buffer, 2.8 µl MgCl_2_ (3.5 mM), 1 µl PCR nucleotide mix (0.5 mM for each deoxynucleoside triphosphate [dNTP]; Thermo Fisher Scientific), 1 µl GoScript reverse transcriptase, and 6.2 µl nuclease-free water. Reactions were annealed at 25°C for 5 min, followed by extension at 42°C for 1 h and reverse transcriptase inactivation at 70°C for 15 min. The cDNA was cleaned using a DNA Clean and Concentrator 5 kit (Zymo Research) according to the manufacturer’s recommendations.

Metagenomic DNA and cDNA were used as templates for PCR amplification of hypervariable region V4 of the bacterial 16S rRNA gene, using Phusion High-Fidelity PCR Master Mix (Thermo Fisher Scientific) and Golay-barcoded primers 515F and 806R adapted from Caporaso et al. (60), including internal spacers of 0 to 7 bp in length adapted from the method described by Fadrosh et al. (61). See Table S3 for primer, barcode, and spacer sequences.

10.1128/mSystems.00262-18.9 TABLE S3 Primer sequences and the bioinformatic scripts and commands that were used. Download Table S3, XLSX file, 0.03 MB. Copyright © 2018 Wurm et al. 2018 Wurm et al. This content is distributed under the terms of the Creative Commons Attribution 4.0 International license.

PCRs were carried out with reaction mixtures containing 10 μl 2× Phusion Master Mix, 5 μl of each of the primers (1.6 μM final concentration), 0.6 µl dimethyl sulfoxide (DMSO), and 4.4 μl template DNA at 98°C for 2 min, with 30 cycles at 98°C for 10 s, 52°C for 15 s, and 72°C for 15 s and a final extension at 72°C for 5 min.

Equimolar amounts of all PCR products were prepared using SequalPrep normalization plate kit 96 (Thermo Fisher Scientific) and were pooled and concentrated using a DNA Clean and Concentrator 5 kit (Zymo Research). Sequencing libraries were prepared with a NEBNext Ultra DNA library preparation kit (New England Biolabs) and sequenced following the manufacturer’s recommendations on an Illumina MiSeq instrument (MiSeq Reagent kit v3; 600 cycles) at the University of Hohenheim, Hohenheim, Germany.

### Quantitative 16S rRNA gene and transcript amplifications.

To determine bacterial loads in human aspirate and mouse stomach samples, metagenomic DNA and cDNA were diluted 1:100 and duplicates of 2 µl used as the template for quantitative PCR with a Femto bacterial DNA quantification kit (Zymo Research), according to the manufacturer’s recommendations. Genomic DNA from E. coli strain JM109, which is part of the kit and contains seven 16S rRNA gene copies per genome, was used as an internal standard to estimate bacterial 16S rRNA gene or transcript copy numbers. The reactions were run on a CFX96 Touch real-time detection system (Bio-Rad). See Table S2 for protocol details. Samples were considered negative if the quantification cycle number was greater than 39. An average quantification cycle (*C_q_*) value was calculated for each sample and used for determination of the numbers of 16S gene copies and transcripts. The estimated bacterial 16S rRNA gene or transcript copy numbers were calculated as averages from the two technical replicates and normalized to 1 ml of human gastric aspirate and the entire stomach of one mouse (Table S2).

### Microbiota analysis and statistical methods.

Raw sequence data from the Roche/454 GS FLX Titanium platform were processed with Mothur (62) according to the standard operating procedure for 454 sequence data (https://mothur.org/wiki/454_SOP [63]) with additional removal of singletons. Subsequent OTU-based microbiota analyses were performed in QIIME v1.8.0 (64), including OTU clustering with 97% similarity, rarefaction to a sampling depth of 1,415 reads (human aspirate, gerbil and mouse samples) or 880 reads (all human and animal samples) per sample, and alpha- and beta-diversity analyses. Differences in taxonomic microbiota compositions between groups were analyzed with the linear discriminant analysis effect size (LEfSe) algorithm (65). For the proof-of-concept study, raw sequence data from the Illumina MiSeq platform were preprocessed with QIIME v. 1.9.1 and cutadapt (66), including extraction of barcodes, merging of read pairs, and demultiplexing and trimming of spacers and primers. OTUs were generated based on a similarity threshold of 95%, and samples were rarefied to 10,000 reads per sample.

The specific statistics tests used to determine significance are listed in figure legends. *P* values below 0.05 were considered significant (*, *P* < 0.05; **, *P* < 0.01; ***, *P* < 0.001). Unless indicated differently, mean values are presented together with standard errors of the means (SEM). Detailed information about all bioinformatic scripts and commands used is provided in Table S3.

### Ethics approval and consent to participate.

The Institutional Review Board of the Medical University of Graz approved the study under protocol number EK 23-212 ex 10/11, and all subjects provided written informed consent to participate in the study. All experimental procedures related to the Mongolian gerbils were carried out at the Ludwig-Maximilians-University of Munich in accordance with the German Law of Animal Welfare and approved by the Regierung of Oberbayern, Oberbayern, Germany (AZ 55.2-1-54-2532-1-2011). Procedures performed on mice were approved by the Institutional Animal Care and Use Committee of the University of Maryland in Baltimore (protocol 0812002) or were carried out at the University of Hohenheim in accordance with the German Law of Animal Welfare.

### Availability of data and material.

Raw sequence files have been deposited in the European Nucleotide Archive under primary accession number PRJEB11744 (secondary accession number ERP013156).
