# Supplementary figures and images for: Qualitative and Quantitative DNA- and RNA-Based Analysis of the Bacterial Stomach Microbiota in Humans, Mice, and Gerbils
Source: mSystems. 2018 Nov 20;3(6):e00262-18. doi: 10.1128/mSystems.00262-18 (PMC6247015; doi:10.1128/mSystems.00262-18)

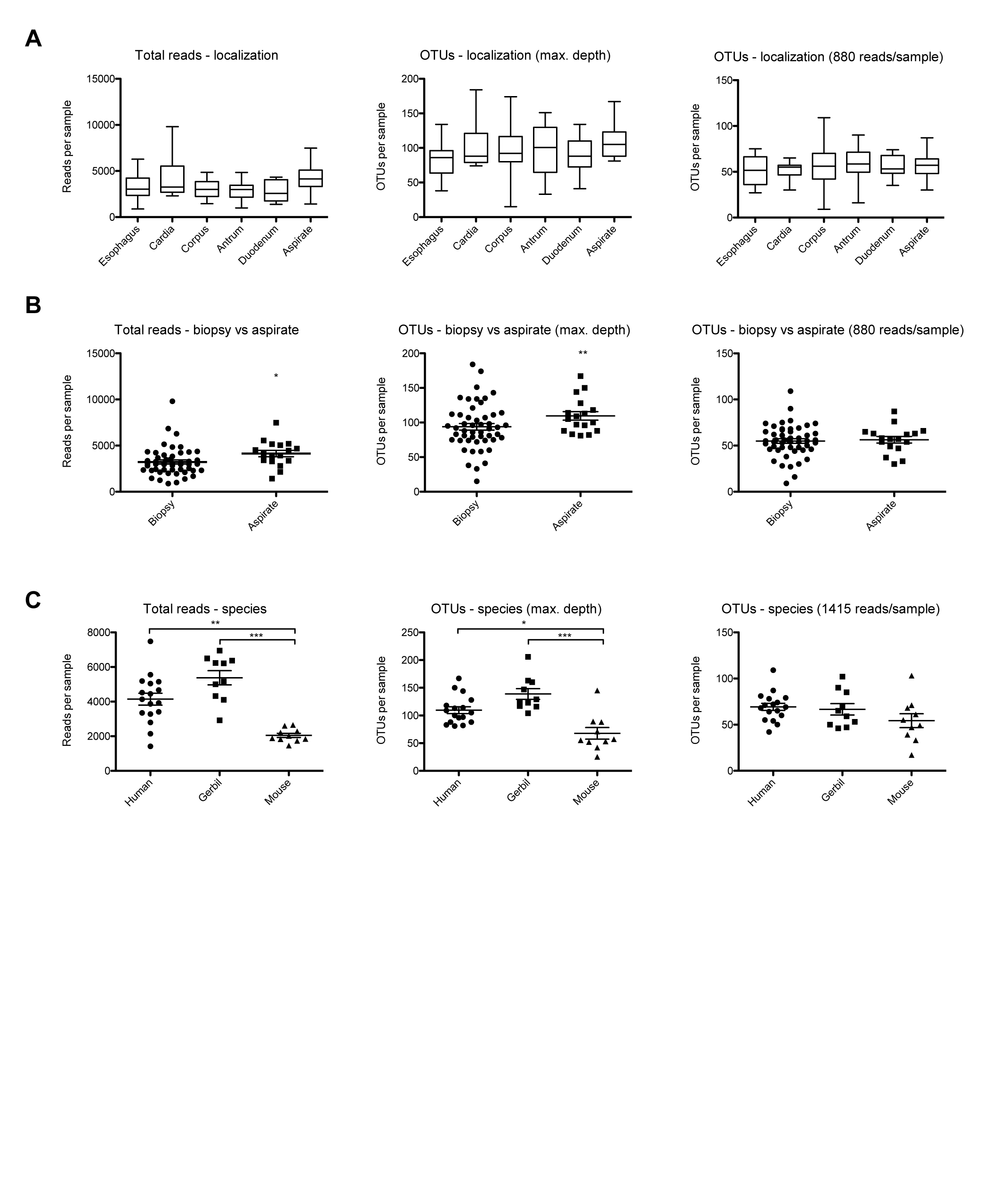

Supplement: FIG S1 [file sys006182292sf1.tif]

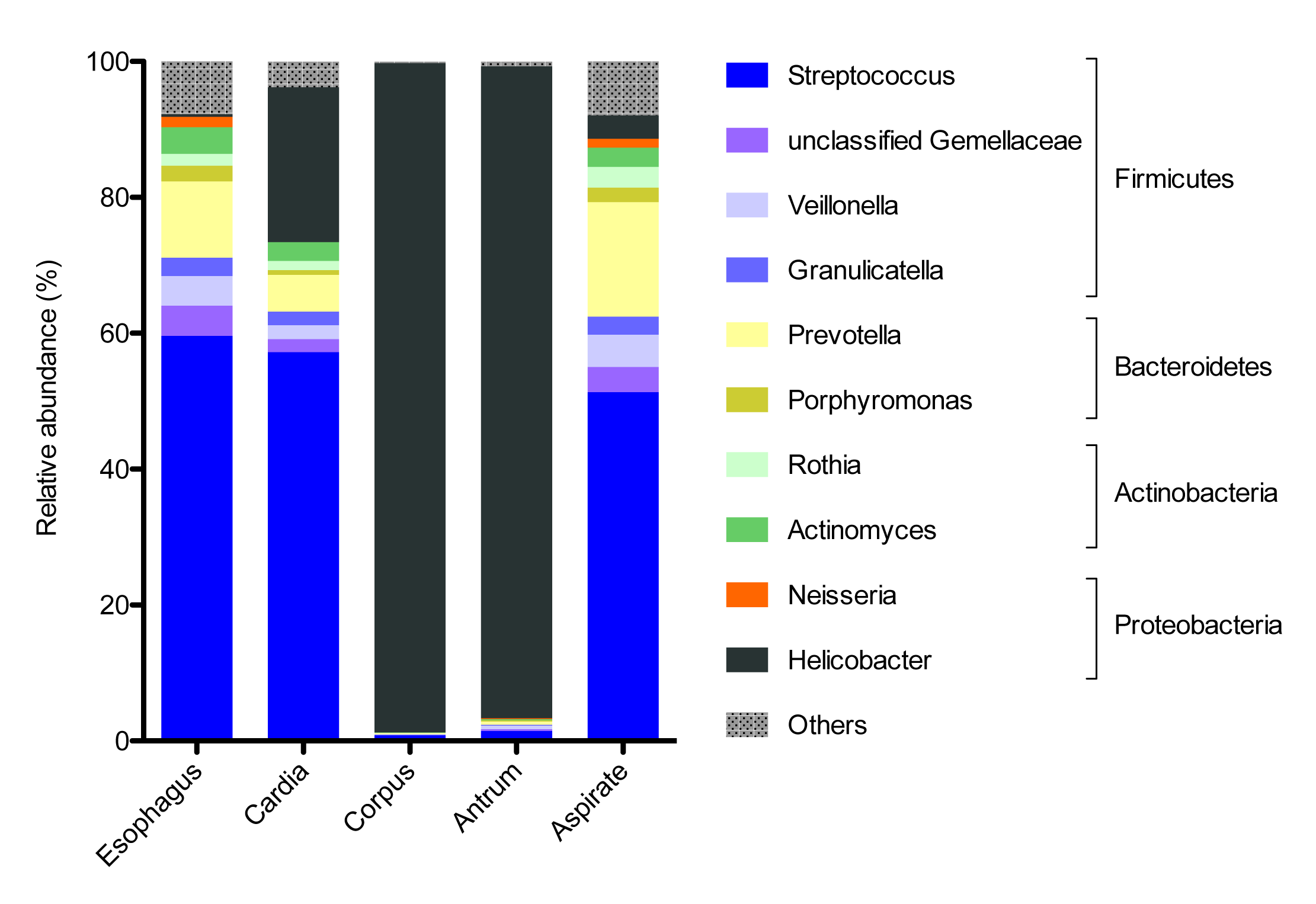

Supplement: FIG S2 [file sys006182292sf2.tif]

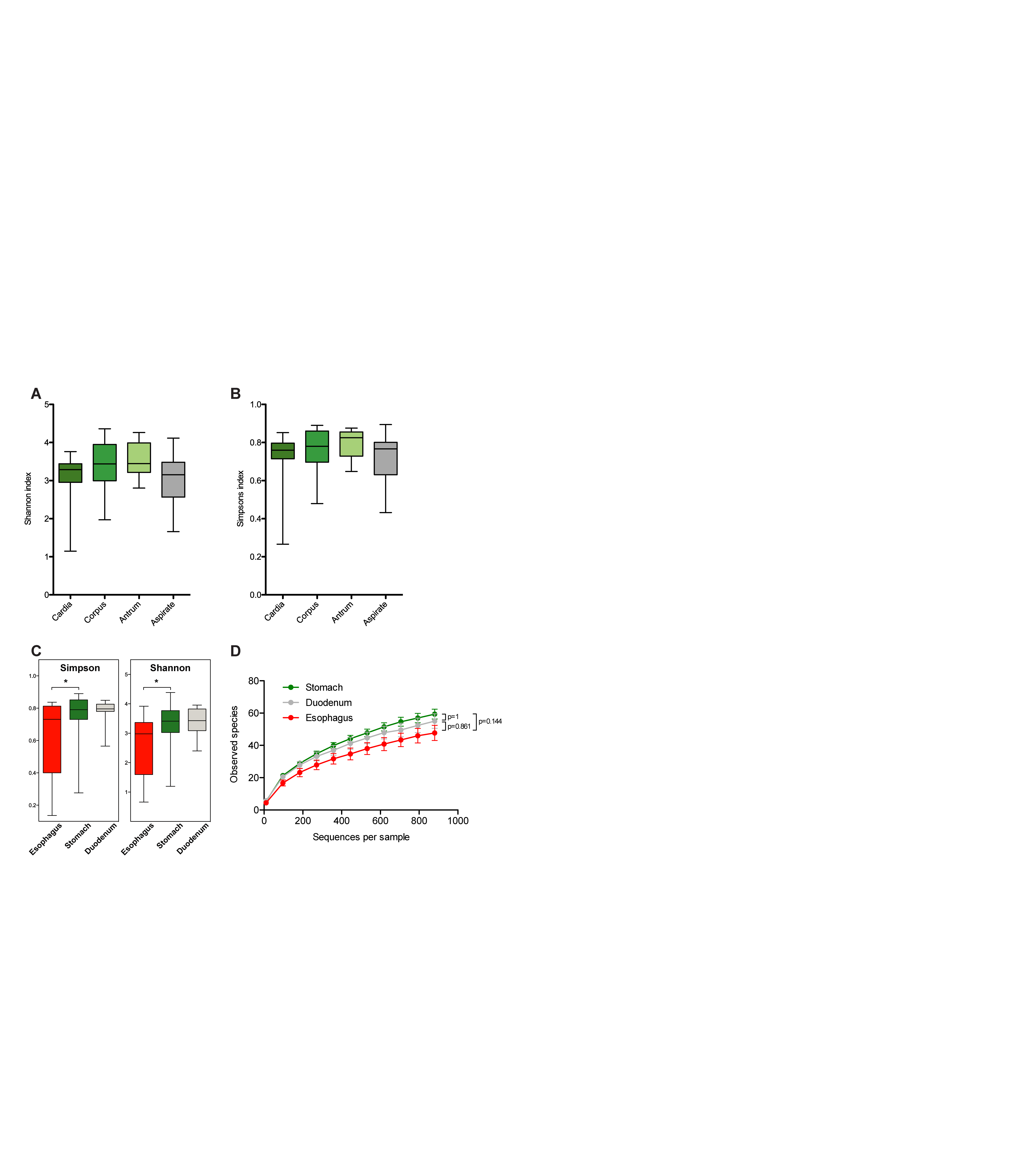

Supplement: FIG S3 [file sys006182292sf3.tif]

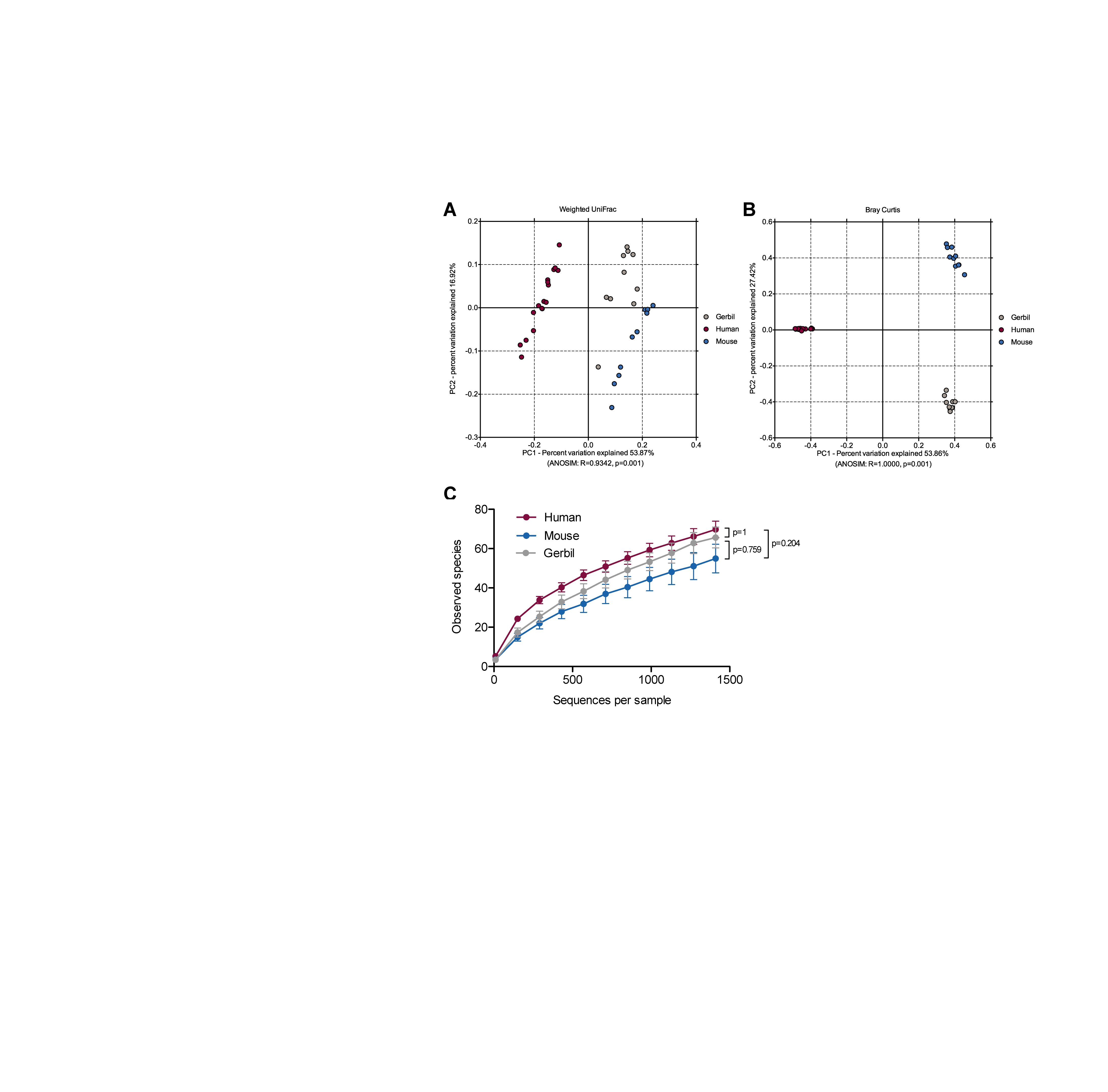

Supplement: FIG S4 [file sys006182292sf4.tif]

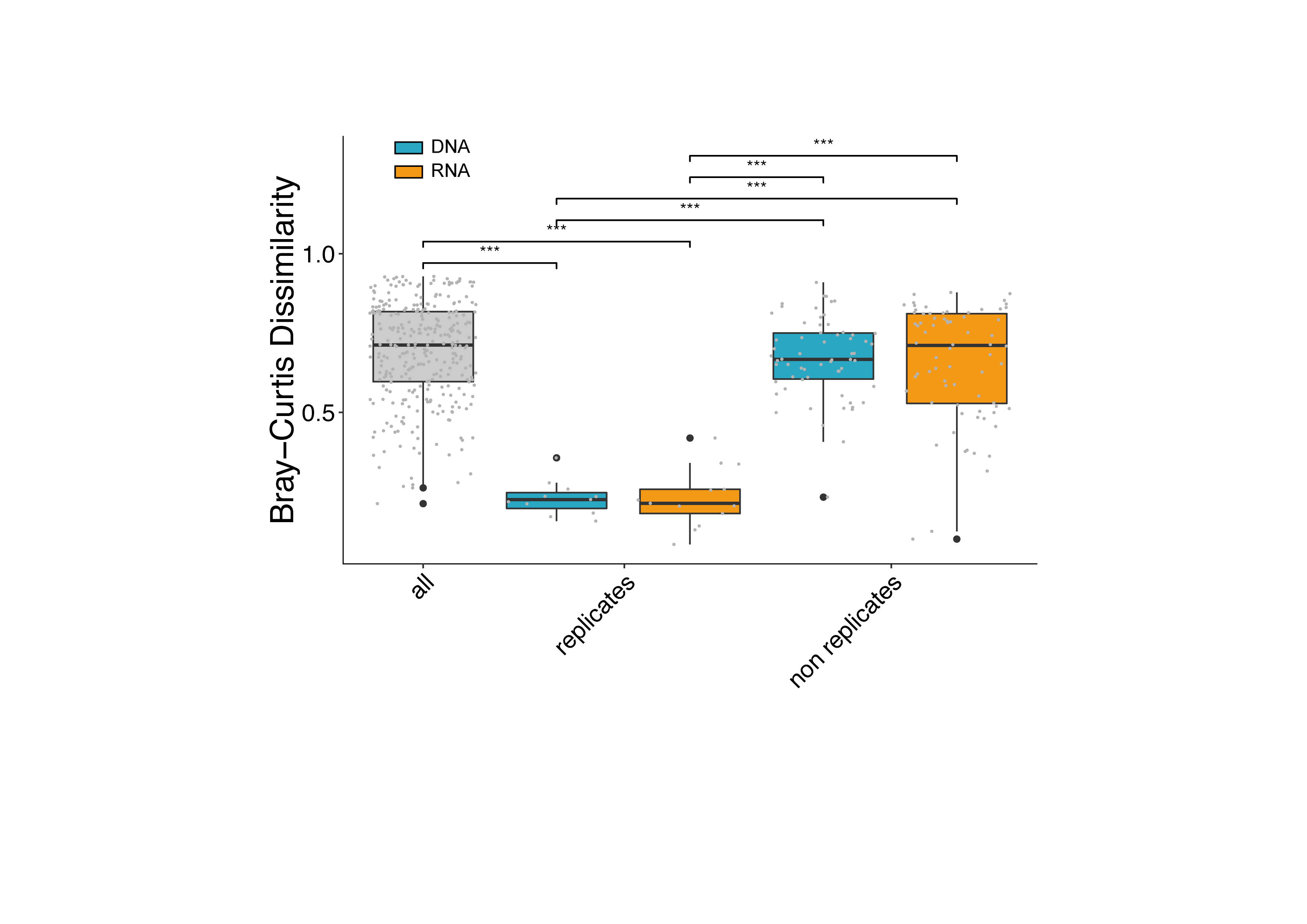

Supplement: FIG S5 [file sys006182292sf5.tif]

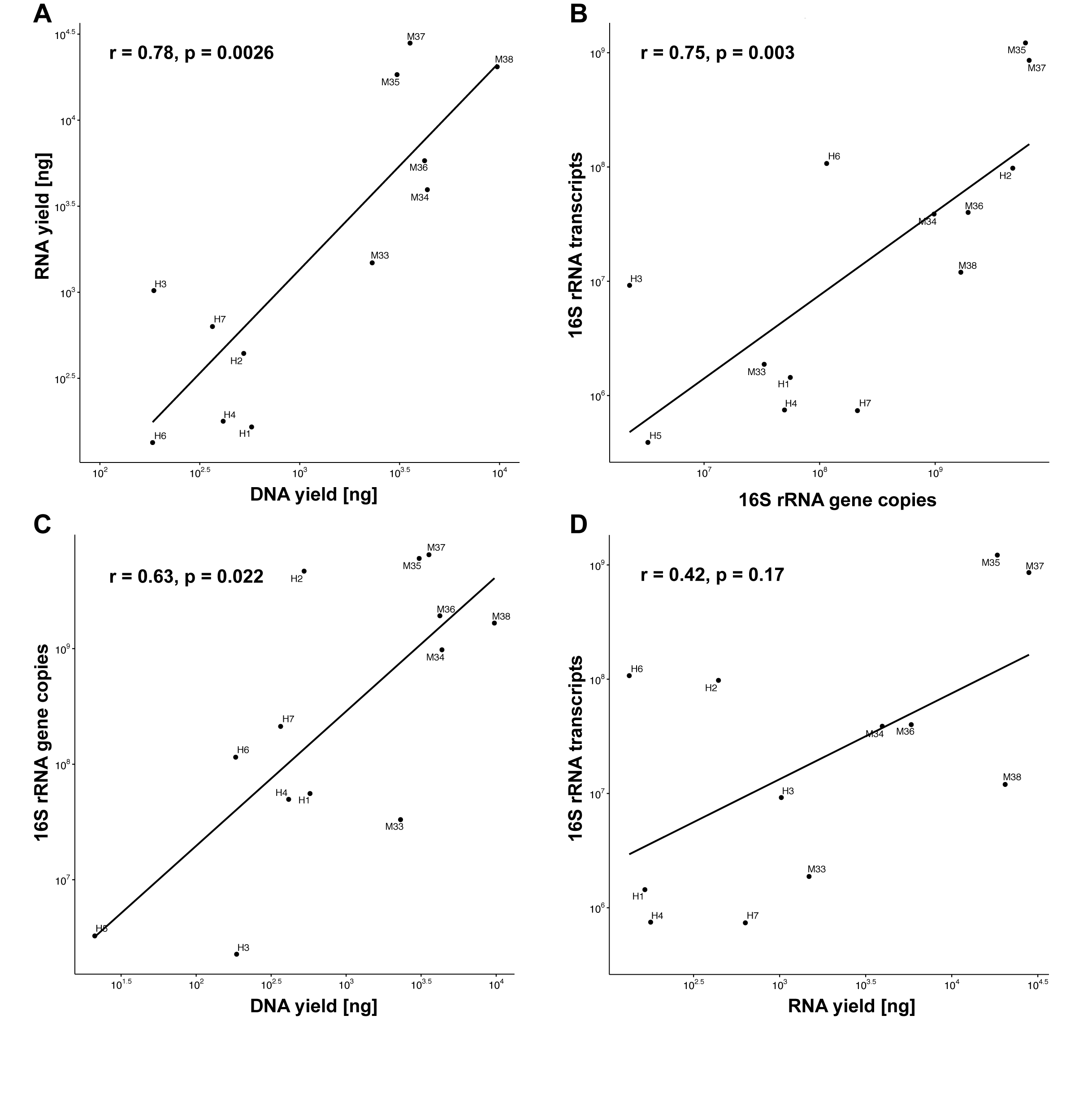

Supplement: FIG S6 [file sys006182292sf6.tif]
